# The Role of *COL1A1*, *COL5A1*, *ACTN3*, *MMP3*, and *GDF5* Gene Variants in Common Sports Injuries: Systematic Review of ACL Rupture, Achilles Tendinopathy, and Stress Fractures

**DOI:** 10.3390/genes17020212

**Published:** 2026-02-09

**Authors:** Shahd Abboud, Elizabeth Akam, David John Hunter, Sarabjit Mastana

**Affiliations:** School of Sport, Exercise and Health Sciences, Loughborough University, Loughborough LE11 3TU, UK; em-elaboud@hotmail.com (S.A.); e.c.akam@lboro.ac.uk (E.A.); d.j.hunter@lboro.ac.uk (D.J.H.)

**Keywords:** sport injuries, ACL rupture, Achilles tendinopathy, stress fractures, genetics, *COL1A1*, *COL5A1*, *MMP3*, *ACTN3*, *GDF5*

## Abstract

Background: Anterior cruciate ligament (ACL) rupture, Achilles tendinopathy, and stress fracture are common sports injuries with significant long-term effects on performance and health. Despite similar exposure, injury susceptibility varies among athletes, suggesting a genetic component. Variants in *COL1A1*, *COL5A1*, *ACTN3*, *MMP3*, and *GDF5* genes influence collagen integrity, muscle performance, and extracellular matrix remodelling, making them potential risk factors. Objective: To systematically review associations between five selected genes and musculoskeletal injury risk. Methods: Following PRISMA 2020 guidelines, PubMed, EMBASE, SPORTDiscus, and Web of Science were searched for studies examining these genes in relation to sports injuries. Data were extracted using Covidence and assessed for quality via the Newcastle–Ottawa Scale (NOS). Results: Twenty-six studies (n > 7000) were included. *COL1A1* rs1800012 showed a protective effect against ACL rupture; *COL5A1* rs1272 and rs13946 increased risk for ACL rupture and Achilles tendinopathy. *MMP3* variants (rs679620, 5A/6A) showed variable associations, particularly in combination with *COL5A1. ACTN3* R577X was linked to higher muscle and soft tissue injury risk in XX genotype carriers. Evidence for *GDF5* rs143383 was limited but suggested a possible association with stress fractures. Conclusions: Genetic variants in *COL1A1*, *COL5A1*, *MMP3*, *ACTN3*, and *GDF5* may influence susceptibility to ACL rupture, Achilles tendinopathy, and stress fractures. Larger, multi-ethnic studies are needed to validate these findings and inform personalised injury prevention strategies.

## 1. Introduction

Sport-related musculoskeletal injuries are a major concern across all levels of athletic participation, causing a substantial burden on performance, career longevity, and long-term health. High-impact injuries such as anterior cruciate ligament (ACL) ruptures, Achilles tendinopathy, and stress fractures are very common and can lead to lengthy rehabilitation, impaired athletic performance, or even premature retirement from sport [1,2,3]. Traditionally, injury prevention initiatives have focused on identifying extrinsic and intrinsic factors that contribute to an athlete’s risk profile [3,4,5,6]. Extrinsic factors encompass external conditions encountered during training or competition, including abrupt increases in training volume or intensity, playing surface characteristics, inappropriate footwear, and environmental conditions. These factors directly influence mechanical loading patterns and may alter movement biomechanics, particularly under fatigue [5]. Conversely, intrinsic factors reflect individual characteristics such as biomechanical alignment, muscular strength deficits, joint laxity, and neuromuscular control. Impairments in proprioception or delayed muscle activation can compromise protective reflexes around joints, thereby increasing susceptibility to injury. A prior history of musculoskeletal injury constitutes a critical intrinsic risk factor, as residual weakness, altered movement mechanics, and incomplete tissue recovery may predispose athletes to reinjury [6]. Although these traditional risk factors are well established, they do not fully explain the pronounced inter-individual variability in injury susceptibility observed among athletes. Some individuals appear inherently more “injury-prone” despite optimal conditioning and technique, while others remain relatively “injury-resistant” even under comparable biomechanical and environmental demands. This variability has contributed to growing interest in the role of genetic predisposition in determining sports injury risk [7,8,9,10,11].

The rapid evolution of sports genomics over the past two decades—driven by advances in molecular genetics, genome-wide association studies (GWAS), and high-throughput genotyping technologies—has enabled researchers to identify genetic variants associated with both performance characteristics and musculoskeletal injury susceptibility [8,9,10]. Of particular interest are functional polymorphisms, which represent common variations in the DNA sequence that may alter protein structure or expression. These variants can influence key biological processes, including collagen synthesis and organisation, tendon elasticity, muscle fibre function, and inflammatory responses, ultimately shaping tissue resilience, mechanical integrity, and recovery capacity [10]. Understanding these genetic contributions may help explain why athletes with similar training histories and biomechanical profiles encounter different injury outcomes. Anterior cruciate ligament (ACL) rupture, Achilles tendinopathy and stress fracture are the most common injuries. Each is influenced by a range of mechanical, anatomical, and environmental factors [1,2,3,4,5,6,12,13,14,15,16,17,18,19,20,21,22,23,24,25,26].

Table 1 summarises the typical injury mechanisms and evidence-based risk factors for the three focal injuries of this review, along with the genetic markers identified.

Genetic variants that influence tissue repair, collagen structure, tendon stiffness, or inflammatory signalling may interact with behavioural and environmental factors—such as training load or nutritional status—to shape an athlete’s injury profile [27,28,29]. These interactions likely contribute to the heterogeneity in injury outcomes even among athletes with similar exposure levels. This systematic review focuses on five key genetic polymorphisms (*COL1A1*, *COL5A1*, *ACTN3*, *GDF5*, and *MMP3*) that have shown significant associations with musculoskeletal sport injuries. Each of these genes plays a distinct role in the structure or function of connective tissues and muscle, and thus each may contribute to injury risk in different ways [9,10,11]:

• *COL1A1* (Collagen Type I Alpha 1 Chain): Encodes a subunit of type I collagen, the primary structural protein found in ligaments, tendons, and bone. A well-studied variant in this gene (the rs1800012 polymorphism in the Sp1-binding site) alters collagen fibril properties, which can reduce tissue tensile strength. As a result, this variant has been associated with an increased risk of soft tissue injuries, such as ligament tears and tendon ruptures [16,17].
• *COL5A1* (Collagen Type V Alpha 1 Chain): This gene encodes a component of type V collagen, which serves as a regulator of collagen fibril formation and organisation in connective tissue. The rs12722 polymorphism of *COL5A1* has been associated with variability in tissue flexibility and stiffness, and it is known to affect one’s susceptibility to tendinopathy and ligament injuries. In particular, this polymorphism is associated with a greater prevalence of Achilles tendinopathy and ACL ruptures, suggesting that collagen V reflects a person’s ability for structural tissue integrity and resilience to injury in tendons and ligaments [30].
• *ACTN3* (Alpha-Actinin-3): Encodes α-actinin-3, a protein predominantly present in fast-twitch (type II) muscle fibres, which serve an important role in the performance of fast, explosive movements. A common polymorphism in the gene, R577X, either permits the presence (R allele) or absence (X allele) of the functional protein α-actinin-3. The absence of α-actinin-3 (XX genotype) changes muscle fibre composition and may compromise power output and fatigue resistance. Genotype XX has been associated with increased risk for specific musculoskeletal injuries, particularly those incurred while participating in sports that require powerful sprinting, jumping, or rapid directional changes, in which protection by fast-twitch fibres is critical [31].
• *GDF5* (Growth Differentiation Factor 5): Encodes a growth factor that plays an important role in joint development, maintenance of cartilage, and tissue repair. Polymorphisms in *GDF5* (e.g., rs143383) reduce the production of growth factor and impede cartilage formation and repair. The genetic variant is well characterised for its association with increased risk of osteoarthritis in weight-bearing joints, which leads to the inference that there is a higher risk of joint injuries and increased recovery time from tissue damage [32].
• *MMP3* (Matrix Metalloproteinase-3): Encodes an enzyme that degrades extracellular matrix components and is active in tissue remodelling and repair after injury. A functional polymorphism in the *MMP3* gene promoter (commonly referred to as the 5A/6A polymorphism) influences the level of MMP3 expression. The high-activity variant can lead to more aggressive matrix breakdown, whereas the low-activity variant might slow tissue remodelling. These differences in MMP3 activity have been linked to variability in recovery rates and injury severity—for example, certain alleles are associated with a greater risk of tendinopathies and ligament damage due to either insufficient repair or excessive degradation of collagen fibres [33].

This systematic review aims not only to summarise the evidence but also to explore the potential for clinical applications in injury prevention. The review seeks to contribute to the advancement of personalised sport genomics and the development of targeted strategies to reduce injury risk among athletes.

## 2. Materials and Methods

Study Design: This systematic review study was conducted following the Preferred Reporting Items for Systematic Reviews and Meta-Analyses (PRISMA) 2020 guidelines [34].

Studies were selected based on predefined inclusion and exclusion criteria structured using the PICOS framework:

Inclusion Criteria

Population: Athletes of all ages, sexes, and competition levels, and physically active individuals.

Intervention/Exposure: Genetic polymorphisms (*COL1A1*, *COL5A1*, *ACTN3*, *GDF5*, or *MMP3*) or genome-wide signals assessed in relation to athletic performance, physiological traits, or injury outcomes.

Comparator: Athletes with different genotypes, or athletes compared with non-athlete control populations when relevant.

Outcomes: Injury incidence, severity, or recovery.

Study Designs: Observational studies (case–control and cohort), candidate-gene studies, and genome-wide association studies (GWAS) and meta-analyses.

Exclusion criteria included: (1) studies not involving athletes or sports injuries; (2) studies not involving human genetic data; (3) reviews, commentaries, editorials, or conference abstracts without primary data; (4) animal or in vitro studies; and (5) studies lacking extractable results.

Data Sources and Search Strategy: PubMed, EMBASE, SPORTDiscus (EBSCOhost), and Web of Science were searched using Boolean combinations of gene symbols and injury terms (e.g., “*COL5A1*” AND “Achilles” OR “tendinopathy”; “*COL1A1*” AND “ACL”; “*ACTN3*” AND “injury”). Reference lists and the relevant grey literature were screened. The time scale for this review was limited to the last 20 years (until 1 August 2025).

**Figure 1:**
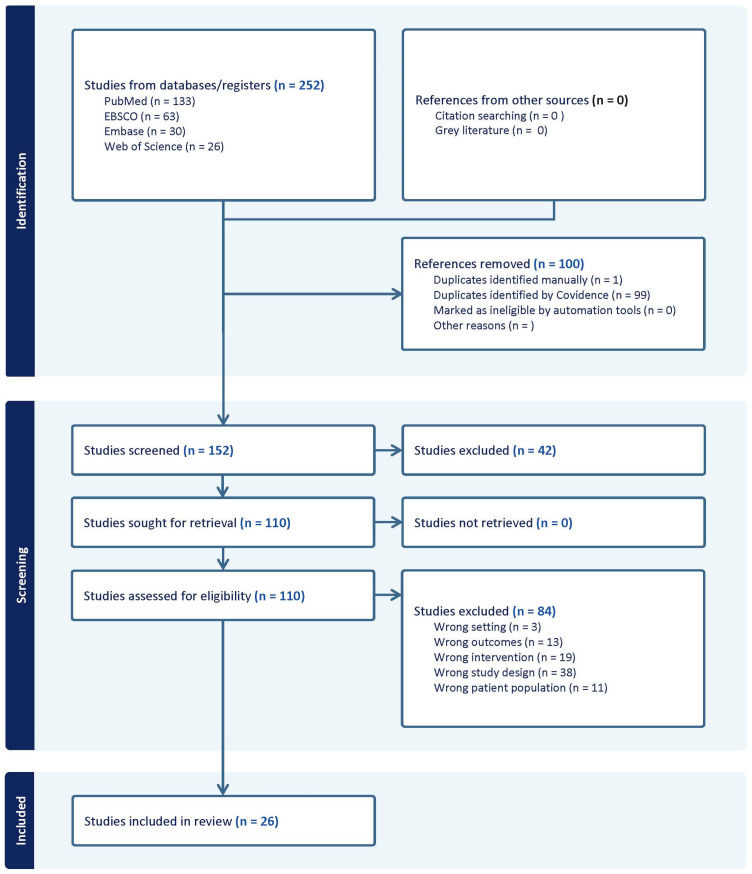
PRISMA Flow diagram for this systematic review (Supplementary Materials).

Study Selection: All search results were imported into Covidence systematic review software 2025 [35], and duplicates were removed. Titles and abstracts were screened against the eligibility criteria by two reviewers. Articles deemed potentially relevant were retrieved in full and assessed independently by two reviewers. Disagreements were resolved through discussion. Following the PRISMA flow diagram (Figure 1), 26 studies were identified that met the eligibility criteria.

Data Extraction: Data from included studies were extracted using a structured framework template on Covidence. A standardised extraction template was developed to ensure consistency across all records. For each eligible study, the following information was systematically recorded: study characteristics (first author, year of publication, country, and study design), population details (athlete status, sample size, and sex), genetic markers (specific genes and single-nucleotide polymorphism investigated), injury focus (type of musculoskeletal injury), outcomes (association between genetic variants and injury risk), and main results. The summary of all studies is presented in Table 2.

Quality Assessment: The Newcastle–Ottawa Scale (NOS) [56] was applied to assess the risk of bias. The NOS evaluates studies across three domains, selection of study groups, comparability of cases and controls or cohorts, and outcome/exposure assessment, with a maximum possible score of nine stars. Higher scores indicate a lower risk of bias. Studies were assessed according to their design using either the case–control or cohort version of the NOS. Each study was checked individually, and if it met the NOS criteria, it was awarded a star. The results of the quality assessment are presented in separate tables for cohort (Table 3) and case–control studies (Table 4). McCabe et al.’s 2018 study [50] is a cross-sectional study; therefore, its NOS rating is very low.

## 3. Results

A total of 26 studies met the inclusion criteria, as shown in the PRISMA flow diagram (Figure 1). These comprised case–control and cohort designs and involved physically active individuals or athletes across a broad range of sports, including football, rugby, athletics, and mixed sporting disciplines (see Table 2). The samples represented diverse populations from Europe, Asia, Australia, South America, and Africa. Among all investigated variants, polymorphisms within collagen-encoding genes (*COL1A1*, *COL1A2*, and *COL5A1*) were the most frequently examined. By contrast, despite its biological relevance, relatively few studies have focused on *GDF5*.

Given heterogeneity across study designs, genetic markers assessed, and injury outcomes, a qualitative synthesis was undertaken. Findings are presented across three primary categories: (1) anterior cruciate ligament (ACL) rupture, (2) Achilles tendinopathy and rupture, and (3) stress fractures and bone-related injuries. A small number of studies examined broader soft tissue or muscle injuries; these are summarised separately. Within each category, results are synthesised on an individual gene/polymorphism basis. Where multiple studies examined the same polymorphism or gene, patterns of association and consistency of evidence are reported. A meta-analysis was not performed due to variability in study design, outcome measures, and reporting standards.

### 3.1. ACL-Related Genetic Association Studies

Several genetic variants have been associated with ACL rupture susceptibility, but the evidence has overwhelmingly implicated collagen genes (*COL1A1*, *COL1A2*, and *COL5A1*) and matrix metalloproteinases (*MMP3*).

#### 3.1.1. *COL1A1*

Several studies have examined the functional rs1800012 polymorphism in the COL1A1 Sp1-binding site. Posthumus et al. [16] reported that the TT genotype was completely absent among 117 South African ACL rupture cases, suggesting a strong protective effect. Collins et al. [55] later corroborated this in a pooled analysis of independent cohorts from South Africa and Sweden. In their dataset—including 350 cruciate ligament ruptures and 581 controls—the rs1800012 TT genotype was rare overall but markedly underrepresented in injured groups. Only 0.3% of cruciate rupture cases carried the TT genotype compared with 4.1% of controls (*p* = 0.0002), reinforcing its protective role. Perini et al. [45] investigated *COL1A2* variants in 146 ACL rupture cases and 192 controls across multiple sports. They found a strong association with rs42524 and rs2621215: the CC genotype of rs42524 had a six-fold risk increase, while the GG genotype of rs2621215 had a four-fold risk increase. More importantly, the presence of *COL1A1* rs1107946 in the GT or TT genotype with *COL1A2* wildtype alleles had a protective effect (OR = 0.25).

#### 3.1.2. *COL5A1*

Posthumus et al. [41] demonstrated a sex-specific effect of the rs12722 polymorphism, with the CC genotype underrepresented (and thus protective) in female ACL cases, while the TT genotype was enriched. O’Connell et al. [44] extended these findings in a large multi-population case–control study involving 333 ACL rupture cases and 378 active controls from South Africa and Poland. rs12722 in *COL5A1* was independently associated with the risk of ACL rupture in female athletes, and the haplotype based on the combination of alleles at the rs12722 of *COL5A1* and rs970547 of *COL12A1* was significantly overrepresented in female cases, thus underscoring the importance of sex-specific gene–gene interactions. Lulińska-Kuklik and colleagues [17] further examined *COL5A1* variants in 134 Polish professional male footballers with surgically confirmed ACL rupture compared to 211 healthy controls. They found no differences for rs12722 or rs13945, but the rs13946 variant showed a significant association in a dominant mode of inheritance (*p* = 0.039). More recently, Rodas et al. [54] analysed 46 elite footballers at FC Barcelona and showed that the rs13946 CC genotype was significantly more frequent in female players with ACL rupture, reinforcing sex-specific susceptibility.

#### 3.1.3. *MMP3*

Two studies evaluated the role of matrix metalloproteinase-3 (*MMP3*) in ACL injuries. Simunić-Briški et al. [47] investigated 95 ACL rupture cases and 92 controls, reporting that risk genotypes rs591058 TT, rs650108 GG, and rs679620 AA were significantly overrepresented in cases. Haplotype analysis identified T–G–A as the risk holotype and C–A–G was protective. Conversely, Malila et al. 2011 [49] examined 86 ACL cases and 100 controls in a Thai cohort, finding no overall effect of the −1612 5A/6A polymorphism, though a subgroup analysis revealed that the 5A+ genotype was more frequent in contact injury cases.

### 3.2. Achilles Tendinopathy (AT)/Rupture Studies

#### 3.2.1. *COL5A1*

Evidence implicates COL5A1 in chronic Achilles pathology. September et al. [21] assessed two independent cohorts (South Africa 93 AT vs. 132 controls; Australia 85 AT vs. 210 controls) for several *COL5A1* variants (including rs12722, rs3196378, rs13946, and others). The rs12722 CC genotype was protective for tendinopathy in both cohorts (AUS OR ≈ 0.42; SA OR ≈ 0.38). In the Australian sample, rs3196378 AC elevated the risk of tendinopathy. Brown et al. [41] (UK case–control study; 112 AT, 227 controls) investigated *COL5A1* rs12722/rs3196378 along with other ECM (extracellular matrix) genes; they reported that the C allele rs12722 was protective for AT, while other variants in the ECM increased risk.

#### 3.2.2. *MMP3*

*MMP3* variants have shown consistent associations with chronic Achilles pathology. Raleigh et al. [22] reported elevated tendinopathy risk among individuals carrying rs679620 GG, rs591058 CC, or rs650108 AA genotypes, and identified a protective ATG haplotype. Briski and colleagues [46] demonstrated a similar pattern in a Croatian athlete sample (63 AT vs. 92 retired athlete controls), where rs650108 GG (OR = 2.46) and rs679620 AA (OR = 3.14) were significantly overrepresented in cases. Recent findings from Brazier et al. [43] extended the evidence base by analysing elite male rugby athletes. This case–control study identified key roles for both *MMP3* rs679620 and *COL5A1* polymorphisms in soft tissue injury susceptibility. The TT genotype of MMP3 rs679620 was significantly overrepresented in players with ligament ruptures and sprains. The T allele appeared protective against tendinopathy. For *COL5A1*, carriers of the C allele at rs12722 were markedly more common in the tendon rupture group compared to non-injured athletes, conferring an eight-fold increased risk. When *COL5A1* rs12722 and rs3196378 were analysed together, the T-C haplotype was enriched in tendon rupture, ligament sprain, and overall injured groups.

### 3.3. Stress Fracture Studies

#### 3.3.1. *COL1A1*

Miyamoto-Mikami et al. [57] analysed a Japanese sample (Stage 1 cross-sectional n = 1667; Stage 2 prospective n = 508) and reported the differential role of rs1107946; the C allele showed female-specific antagonistic effects: higher fatigue fracture risk (OR ≈ 2.4) but lower muscle injury risk (OR ≈ 0.46). Mechanistic profiling suggested that this allele may lower bone mineral density (BMD), reduce muscle–tendon stiffness, and shift collagen composition toward a greater α1-chain homotrimer ratio, indicating a potential bone–muscle trade-off.

#### 3.3.2. *COL5A1*

Evidence from Jacob et al. 2022 [51] shows that the *COL5A1* rs12722 TT genotype increases the risk of bone and stress injuries in elite Australian Football League athletes (7-season prospective AFL cohort; n = 46 with 992 injuries). Varamenti et al. [52] further reported that rs12722 TT was protective for tendon and muscle injuries, emphasising the complex role of type V collagen across tissue types.

#### 3.3.3. *GDF5*

Zhao et al. [25] conducted a prospective cohort study among 1398 Chinese male infantry recruits undergoing eight weeks of basic training. The study reported a 13.5% incidence of radiologically confirmed stress fractures. In addition to established risk factors such as prior fracture history and lower pre-training exercise level, a significant genetic association was observed for the *GDF5* rs143383 polymorphism. The T allele was more prevalent among cases (79.1% vs. 68.4% in controls), conferring a 1.75-fold increased risk (95% CI: 1.35-2.28).

### 3.4. Broader Muscle and Soft Tissue Injuries

Five studies [36,37,38,39,40] from different parts of the world (Brazil, Italy, Spain and Sweden examined the role of the *ACTN3* polymorphism in soft tissue injuries, and in most studies, XX genotype carriers documented a higher risk of injury. The sample sizes are relatively small; therefore, caution is warranted.

## 4. Discussion

This review explored the role of genetic variation in musculoskeletal injury susceptibility alongside established biomechanical and training-related factors. Collagen-related loci (*COL1A1*/*COL1A2*/*COL5A1*) affect fibril structure and tissue mechanics, modulating ligament and tendon resilience. MMP3 variants may alter extracellular matrix turnover, influencing repair dynamics after microtrauma. *ACTN3* loss of function (XX genotype) is consistently linked to higher non-contact muscle and soft tissue injury burden, plausibly via reduced fast-twitch function. *GDF5* variation may contribute to bone stress responses. Overall, the results lend support to the notion that genetic variation is an important determinant of injury susceptibility alongside extrinsic–intrinsic risk factors, including, but not limited to, biomechanics, training loads and exposure to surfaces. It is noteworthy that many of the associations noted were sex-specific, context-dependent or moderated by gene–gene interactions, which reflects the complex polygenic nature of risk of musculoskeletal injury. However, heterogeneity, modest sample sizes, and limited representation of non-Caucasian cohorts constrain inference. Functional validation and broader approaches—polygenic risk scores, GWAS, and gene–environment analyses—are needed to refine effect estimates and translation to practice.

### 4.1. Interpretation of Key Findings of ACL Rupture

The *COL1A1* gene encodes a subunit of type 1 collagen, which is the primary structural protein in ligaments, tendons, and bone, critical for tissue tensile strength [16]. Variants in this gene can alter collagen transcription, protein structure, or post-translational modification, influencing the ligament’s mechanical structure and resilience. This review highlights a protective effect of the *COL1A1* rs1800012 TT genotype against ACL rupture. Studies have reported this genotype to be either absent or significantly underrepresented in individuals with ACL ruptures compared to controls [45,55]. This strong association suggests that individuals carrying the TT genotype may have altered type 1 collagen structure or expression that enhances ligament resistance, potentially making them less susceptible to rupture [43]. Beyond direct effects, gene–gene interactions are also crucial; for instance, the *COL1A1* rs1107946 GT or TT genotype conferred a protective effect when combined with *COL1A2* wildtype alleles, suggesting that the overall integrity and resilience of the ACL’s complex collagenous extracellular matrix is modulated by a combination of genetic factors [45].

The *COL5A1* gene encodes a component of type V collagen, which plays a fundamental role in regulating collagen fibril formation and organisation within connective tissues [30]. Polymorphisms in *COL5A1*, particularly rs12722 and rs13946, are known to influence tissue flexibility and stiffness, critical for ligament function and injury resistance [30]. Consistent findings in several studies link these two variants with increased risk for ACL rupture, often demonstrating sex-specific effects [17,40,41]. The rs12722 TT genotype was frequently overrepresented in female ACL rupture cases, and the rs13946 CC genotype was significantly more common in female elite footballers with ACL injuries [40]. The physiological implication is that variations in *COL5A1* can lead to altered collagen fibril diameter and packing, resulting in ligaments that may be either too stiff or too lax. This can compromise the ACL’s ability to effectively absorb and dissipate mechanical stress, thereby increasing susceptibility to rupture, particularly in female athletes, where additional biomechanical and hormonal factors may interact with these genetic predispositions [40]. A recent meta-analysis by our group [58] showed a significant effect of rs12722 on lower limb musculoskeletal injuries in the recessive model of inheritance (OR = 1.28 (CI 1.03–1.57, *p* = 0.021) and ACL (OR = 1.31 (CI 1.03–1.67, *p* = 0.026).

*MMP3* (matrix metalloproteinase-3 gene) encodes an enzyme vital for the degradation and remodelling of extracellular matrix components, a process crucial for tissue repair and adaptation [33]. Studies have shown variable associations between *MMP3* polymorphisms and ACL rupture risk. Specific genotypes (rs591058 TT, rs650108 GG, and rs679620 AA) and a T-G-A risk haplotype were significantly overrepresented in non-contact ACL rupture cases [47]. This suggests that genetic variations leading to dysregulated MMP3 activity can disturb the delicate balance of collagen synthesis and degradation within the ACL. Excessive MMP3 activity could lead to accelerated breakdown of the collagen matrix, weakening the ligament, whereas insufficient activity might impair proper remodelling and repair following microtrauma [33].

### 4.2. Achilles Tendinopathy/Rupture

Evidence from this review consistently implicates *COL5A1* and *MMP3* genes in susceptibility to Achilles tendinopathy and rupture. COL5A1 plays a fundamental role in regulating collagen fibril organisation and overall tissue mechanics. Dysregulation of type V collagen can lead to altered tissue properties, making tendons more susceptible to repetitive strain and microtrauma [30]. The review indicates that the *COL5A1* rs12722 CC genotype is consistently protective against Achilles tendinopathy, suggesting that this variant may contribute to a more resilient tendon structure capable of withstanding chronic loading [21,41]. However, the role of *COL5A1* is complex and potentially injury-specific because the rs12722 C allele was also reported to be associated with a markedly higher risk of acute tendon rupture in elite male rugby players [42]. Physiologically, these findings suggest that while certain *COL5A1* variants might optimise the collagen fibril structure in a way that protects against the chronic degenerative characteristics of tendinopathy, different genetic alterations or the same allele in a different context might predispose the tendon to acute failure under extreme, sudden loads by affecting its elastic limits or ultimate tensile strength.

The role of MMP3 in extracellular matrix (ECM) degradation and remodelling is crucial for Achilles tendon health, as proper repair and adaptation are essential to withstand high mechanical loads. A few studies show that specific *MMP3* risk genotypes (e.g., rs679620 GG/AA, rs591058 CC, and rs650108 AA) [22,46,48] are associated with an increased risk of Achilles tendinopathy, often with synergistic effects when interacting with *COL5A1* variants [22]. Physiologically, these variants likely lead to a state of chronic, imbalanced extracellular matrix turnover, where excessive collagen breakdown or impaired repair capacity weakens the tendon, making it vulnerable to repetitive microtrauma and the development of tendinopathy. Conversely, one study reported that the T allele of *MMP3* rs679620 is protective against tendinopathy, despite its TT genotype being linked to higher ligament injury risk [42]. This complex pattern suggests that MMP3’s influence is highly dependent on the specific variant, the type of tissue, and the exact nature of the injury (chronic degeneration versus acute rupture), reflecting its diverse roles in tissue homeostasis and repair.

### 4.3. Stress Fracture

Relatively few studies met the inclusion criteria examining stress fractures in athletes and the specific genes reviewed here, especially compared with the larger body of research on the ACL and Achilles. This makes it difficult to draw firm conclusions, but the available evidence highlights potential roles for *COL1A1*, *COL5A1*, and *GDF5*.

As the primary structural protein of bone, *COL1A1* is central to bone strength and elasticity. The rs1107946 C allele shows a striking female-specific effect: increasing fatigue fracture risk while reducing muscle injury risk. This allele has been linked to lower bone mineral density and an altered collagen composition (more α1 homotrimers), making bone more vulnerable to microdamage under repetitive loading [26]. These findings suggest a possible bone–muscle trade-off, where genetic adaptations that benefit one tissue may compromise another tissue.

COL5A1, although known for its role in collagen fibril organisation, also appears relevant to bone integrity. The rs12722 TT genotype was associated with a higher incidence of bone injuries, including stress fractures, in elite athletes [52,58]. This suggests that variants in COL5A1 may reduce the bone matrix’s ability to withstand cumulative stress. However, this was a pilot study with a very small sample size, which limits the strength and generalizability of the conclusion.

Finally, GDF5, a key regulator of cartilage and bone development, has limited but notable evidence linking it to stress fractures. A large prospective cohort study of Chinese infantry recruits reported that the rs143383 T allele increased stress fracture risk by 1.75-fold, likely reducing GDF5 production and impairing bone remodelling [25]. Together, these findings suggest a potential role for genetic variation in bone stress responses, though further large-scale, athlete-focused studies are essential.

### 4.4. Broader Muscle and Soft Tissue Injuries

*ACTN3* encodes α-actinin-3, a structural protein expressed almost exclusively in fast-twitch muscle fibres, which underpin explosive strength and high-velocity movements in sport. A common polymorphism, R577X, determines whether the protein is present (R allele) or absent (X allele). Individuals with the XX genotype lack functional α-actinin-3, leading to shifts in muscle fibre composition and function [31]. Across multiple studies, the XX genotype has been consistently linked to a greater incidence, odds, and severity of non-contact muscle and soft tissue injuries. Physiologically, α-actinin-3 deficiency reduces peak power output and alters fatigue resistance in fast-twitch fibres, diminishing their ability to withstand repetitive high-intensity loading. This may also affect tendons and ligaments [31]. When muscle function is compromised, surrounding connective tissues are exposed to greater mechanical strain, as they must compensate for reduced force absorption and stability. Over time, this imbalance can elevate the risk of secondary injuries such as tendinopathy, ligament sprains, or even ruptures, particularly in sports demanding rapid and powerful movements [59].

### 4.5. Limitations and Future Directions

Although the findings of this review seem promising, the research is still limited and heterogeneous. Most studies focused on small samples; therefore, they are underpowered, and utilised mostly Caucasian/European samples, limiting the possibility for generalisation to larger and more diverse populations.

The current review is also limited in its scope, as there are only a limited number of studies from different geographical areas, most of which had none or only single studies with small samples, limiting our ability to carry out a review/analysis at different levels to assess how genetic/allele frequency variations may affect the risks/injuries.

There is a need for larger, multi-ancestry, multi-country/continental population-based comprehensive studies on different sport modalities and injuries to further this field and use genomic analyses for personalised therapies/exercise prescription.

A meta-analysis was not undertaken due to the high degree of heterogeneity across the included studies, encompassing substantial variation in population characteristics, methodological approaches, genetic markers assessed, and the injury outcomes used to define musculoskeletal phenotypes. Existing meta-analyses of specific genes and aligned variants, such as *COL5A1*, demonstrate that when study designs, phenotype definitions, and analytical models are sufficiently standardised, robust pooled estimates can be generated [58,60]. These findings indicate that, with greater methodological alignment in future research, the contribution of these variants to musculoskeletal injury risk will be more precisely characterised.

Evidence for certain genes, such as GDF polymorphisms in relation to stress fractures in athletes, was relatively limited. While GDF5 is widely recognised for its involvement in joint development, cartilage maintenance, and its association with osteoarthritis [61], research directly linking its variants to stress fractures in athletic populations is limited.

Additionally, most research has focused on single-nucleotide polymorphisms, whereas musculoskeletal injury risk is likely to be polygenic, requiring broader approaches such as genome-wide association studies (GWAS) and polygenic risk scores.

Other gaps include limited exploration of gene–gene and gene–environment interactions and insufficient attention to sex-specific effects. Lastly, while associations have been documented in the literature, functional validations are warranted to provide evidence for an association between genetic variants and biological processes relevant to musculoskeletal injury, such as collagen remodelling after running a marathon, muscle repair, and regulating inflammation. Advancing the evidence in the literature will be valuable in meaningfully interpreting the state of the science as we apply what we know about genetic studies, interventions, and practice, continuing to develop more personalised targeted methods for injury prevention and management.

### 4.6. Clinical Implications and Future Directions

Genetic profiles could complement screening to personalise training load management, neuromuscular conditioning, and recovery protocols. For example, athletes with COL5A1 risk variants for Achilles tendinopathy may benefit from targeted tendon loading and gradual progression; female athletes with COL1A1 variants linked to fatigue fractures may require proactive bone health monitoring, nutrition optimisation, and workload periodisation. Translation must proceed cautiously, given the current evidence limits and ethical considerations.

Importantly, a further methodological challenge lies in quantifying the relative influence—or practical “weight”—of individual genetic variants within the inherently polygenic and multifactorial landscape of sports injuries. Emerging work using cumulative or total genotype scoring illustrates this complexity. Studies demonstrate that injury risk reflects the combined effect of multiple loci rather than the action of single variants in isolation, highlighting the limitations of monogenic interpretations and underscoring the need for integrated polygenic models in future applied practice [10,62].

As research becomes increasingly standardised in phenotype definitions, methodological quality, and analytical frameworks, these polygenic approaches—alongside high-quality meta-analyses on specific markers such as COL5A1 [58,60]—are likely to refine our ability to interpret genetic susceptibility and translate it into meaningful, evidence-based athletic management strategies.

## 5. Conclusions

Variants in *COL1A1*, *COL5A1*, *ACTN3*, *MMP3*, and *GDF5* are implicated in susceptibility to ACL rupture, Achilles tendinopathy, and stress fractures. While genetic profiling alone is not a definitive predictor, integrating genomic insights with biomechanics, training data, and clinical monitoring may enhance personalised prevention and rehabilitation. Larger, diverse cohorts and mechanistic studies are essential before routine implementation.

## Figures and Tables

**Table 1:** Summary of risk factors for three common sport injuries.

Injury Type	Mechanism	Intrinsic Risk Factors	Extrinsic Risk Factors	Genetic Risk Loci Identified
ACL Rupture	Non-contact pivoting, deceleration, or awkward landing movements [12]	Female sex (≈2–8× higher risk), generalised joint laxity, anatomical differences (e.g., narrow intercondylar notch, increased Q-angle) [13,14]	Fatigue-induced neuromuscular control deficits, poor shoe–surface traction, high sport-specific loading [15]	*COL1A1*, *COL5A1* [16,17]
Achilles Tendinopathy	Repetitive strain and microtrauma to the Achilles tendon [18]	Prior tendon injury, lower-limb biomechanical deviations (e.g., overpronation), reduced plantar-flexor strength, elevated BMI [19]	Rapid training load increase, hard surfaces, inadequate footwear, fluoroquinolone exposure [20]	*COL5A1*, *MMP3* [21,22]
Stress Fracture	Cumulative bone overload [23]	Low bone mineral density, menstrual dysfunction (female athlete triad), low lean body mass, history of stress fractures [24]	Overtraining, insufficient recovery, poor nutrition, rigid training surfaces, RED-S [24]	*COL1A1*, *GDF5* [25,26]

**Table 2:** Summary information for all studies included in this review.

Study	Country	Athlete Status	Design	Sample Size	Gene/SNP	Outcome	Main Results
Clos et al. 2019 [36]	Spain/ Sweden	Elite professional footballers	Prospective cohort	N = 43 (19 RR, 21 RX, 3 XX)	*ACTN3* rs1815739 (R577X)	Non-contact musculoskeletal soft tissue injury rate	XX genotype: 2.78 injuries/season vs. RR 1.51; XX had higher injury incidence.
Del Coso et al. 2022 [37]	Spain	Professional women footballers	Cross-sectional cohort + follow-up	N = 191	*ACTN3* rs1815739 (R577X)	Non-contact injuries; RTP; performance tests	No significant differences in injury incidence across genotypes.
Rodas et al. 2021 [38]	Spain	Elite male and female footballers	Retrospective cohort	N = 46	*ACTN3* rs1815739 (R577X)	Muscle injury incidence; days to injury	XX genotype: all injured ≥ 1×; RR and RX had more injury-free players.
Massidda et al. 2019 [39]	Italy	Professional male footballers	Case–control	N = 257 cases, 265 controls	*ACTN3* rs1815739 (R577X)	Number of muscle injuries; severity; time-loss	XX genotype: 2.66× higher odds of injury vs. RR.
de Almeida et al. 2022 [40]	Brazil	Male professional footballers	Observational cohort	N = 83	*ACTN3* rs1815739; ACE I/D	Injury incidence; severity	*ACTN3* XX: ↑ severe injury risk (OR = 5.14, *p* = 0.007).
Zhao et al. 2016 [25]	China	Male infantry recruits, 8-week basic training	Prospective cohort	1398 (189 stress fractures, 1209 controls)	*GDF5* rs143383	Stress fractures (metatarsal, tibia, femur, pelvis, femoral neck)	Stress fracture incidence was 13.5%. T allele of *GDF5* rs143383 was significantly more frequent in cases (79.1% vs. 68.4%; OR 1.75, 95% CI 1.35–2.28).
Posthumus et al. 2009 [41]	South Africa	Physically active (mixed)	Case–control	N = 129 ACL cases, 216 controls	*COL5A1* rs12722 (C/T)	ACL rupture	Females: CC underrepresented (protective); TT overrepresented in cases.
Brown et al. 2017 [42]	UK	Mixed (active individuals)	Case–control	N = 112 Achilles cases, 227 controls	*COL5A1* rs12722, rs3196378 + ECM	Achilles tendinopathy	*COL5A1* rs12722 C allele protective; ECM variants also implicated.
Lulińska-Kuklik et al. 2018 [17]	Poland	Professional male soccer players	Case–control	134 ACLR cases, 211 controls	*COL5A1* rs12722 (C/T), rs13946 (C/T)	ACL rupture (non-contact)	No general differences for rs12722/rs13946. rs13946 is significant under the dominant model (*p* = 0.039). C–C haplotype protective (overrepresented in controls, *p* = 0.038).
Brazier et al. 2025 [43]	UK/Ireland/South Africa	Elite male rugby players	Case–control	N = 184	13 SNPs incl. *COL5A1* rs12722, rs3196378; *MMP3* rs679620, rs591058, rs650108; plus *COL1A1*, *COL3A1*, *COLGALT1*, *KDR*, *MIR608*, *NID1*, *TIMP2*, *VEGFA*	Tendon rupture, tendinopathy, ligament sprain & rupture	*MMP3* rs679620 TT genotype higher risk of ligament injury (rupture/sprain); the T allele is protective of tendons. *COL5A1* rs12722 C allele has higher risk of tendon rupture (OR = 8.3). *COL5A1* rs12722–rs3196378 T-C haplotype has higher risk across tendon rupture, ligament sprain, and total injured athletes.
Posthumus et al. 2009 [16]	South Africa	Physically active Caucasians	Case–control	N = 117 ACL cases, 130 controls	*COL1A1* rs1800012	ACL rupture	TT genotype absent in cases (protective); family history ↑ risk.
O’Connell et al. 2015 [44]	South Africa & Poland.	Physically active Caucasians	Case–control	333 ACL rupture cases/378 controls	*COL5A1* rs12722, *COL12A1* rs970547, *COL3A1* rs1800255, *COL6A1* rs35796750	ACL rupture	Variants in *COL5A1 rs12722* and *COL12A1* rs970547 are independently associated with ACL rupture risk in females; the combined *COL5A1–COL12A1* haplotype is significantly more frequent in female cases.
Perini et al. 2022 [45]	Brazil	Competitive athletes (mixed sports)	Case–control	N = 338 total (146 ACL, 192 controls)	*COL1A1* rs1107946; *COL1A2* rs412777, rs42524, rs2621215	ACL rupture	*COL1A2* rs42524 CC (≈6×) & rs2621215 GG (≈4×) ↑ non-contact ACL risk; *COL1A1* rs1107946 protective only with *COL1A2* wildtype.
Miyamoto-Mikami et al. 2021 [26]	Japan	Elite & collegiate athletes	Two-stage genetic + prospective	Stage 1 n = 1667; Stage 2 n = 508	*COL1A1* rs1107946	Fatigue fracture; muscle injury; BMD; stiffness	Females: C allele ↑ fatigue fracture (OR ≈ 2.4) and ↓ muscle injury (OR ≈ 0.46); ↓ BMD & stiffness; ↑ α1 homotrimers.
Raleigh et al. 2009 [22]	SA/UK	Active adults	Case–control	75 AT, 39 rupture, 98 controls	*MMP3* rs679620/rs591058/rs650108; *COL5A1* rs12722	Achilles tendinopathy & rupture	AT: rs679620 GG, rs591058 CC, rs650108 AA ↑ risk; ATG haplotype protective; *COL5A1* T + MMP3 G ↑ risk; no rupture effect.
Briški et al. 2021 [46]	Croatia	High-level athletes (cases) & retired athletes (controls)	Case–control	63 AT vs. 92 controls	*MMP3* rs591058/rs650108/rs679620	Achilles tendinopathy	rs650108 GG (OR = 2.46) & rs679620 AA (OR = 3.14) ↑ risk; C–A–G haplotype protective.
Simunic-Briski et al. 2024 [47]	Croatia	High-level athletes & retired athletes	Case–control	95 ACL vs. 92 controls	*MMP3* rs591058/rs650108/rs679620	Non-contact ACL rupture	Risk genotypes: rs591058 TT, rs650108 GG, rs679620 AA; T–G–A haplotype risk; C–A–G protective.
Gibbon et al. 2017 [48]	Australia & South Africa	Recreationally active; matched controls/cases	Case–control genetic association study	Australia: 200 control, TEN: 85 South Africa: 232 CON, 234 ACL	*MMP3:* rs679620 (A/G), rs591058 (T/C), rs650108 (G/A), rs3025058 (5A/6A promoter)	Achilles tendinopathy, ACL rupture	South Africa: rs3025058 (6A allele, linked to rs679620) ↑ risk of Achilles tendinopathy (OR 2.88, 95% CI 1.4–6.1, *p* = 0.012). Australia: 6A-G-C-G haplotype ↓ risk (29% CON vs. 20% TEN, *p* = 0.037). No associations with ACL rupture.
Malila et al. 2011 [49]	Thailand	Active adults	Case–control	86 ACL vs. 100 controls	*MMP3* −1612 5A/6A	ACL rupture	No overall effect; contact-ACL cases had higher 5A+ (OR = 2.25).
McCabe et al. 2018 [50]	UK	Professional, semi-pro & amateur soccer	Cross-sectional (season follow-up)	N = 289	*GDF5* rs143383; *AMPD1* rs17602729; *COL5A1* rs12722; IGF2 rs680	Ankle & knee injuries; availability	*GDF5* TT & *IGF2* GG ↑ injuries; *AMPD1* CC & *COL5A1* TT protective.
Jacob et al. 2022 [51]	Australia	Elite male AFL players	7-season prospective cohort	N = 46; 992 injuries	*COL5A1* rs12722; *COL1A1* rs1800012; *NOGGIN*; *IGF2*, etc.	Muscle, tendon, ligament, bone injuries	*COL5A1* TT ↑ muscle & bone injuries; COL1A1 TT ↑ ligament injuries; *NOGGIN* GG ↑ muscle; *IGF2* CC ↑ tendon injuries.
Varamenti et al. 2024 [52]	Qatar (Arab origin)	High-level male athletes	Pilot cohort/case–control	N = 30	*COL5A1* rs12722; rs10735810	Soft tissue & stress fractures	*COL5A1* TT protective for muscle/tendon; *VDR* CT/TT ↑ stress-fracture risk; TT ↑ severe fractures.
September et al. 2009 [21]	SA & Australia	Active adults	Case–control	SA: 93 AT vs. 132 ctrls; AUS: 85 AT vs. 210 ctrls	*COL5A1* rs12722; rs13946; rs3196378 (and others)	Chronic Achilles tendinopathy	rs12722 CC protective (AUS OR = 0.42; SA OR = 0.38). rs3196378 AC ↑ risk (AUS). SA haplotype TC ↑ risk.
Brazier et al. 2023 [53]	UK/Ireland/South Africa	Elite male rugby vs. non-athletes	Case–control (polygenic)	RA: n = 663, NA: n = 909	13 SNPs incl. *COL5A1* rs12722/rs3196378; *MMP3*; *VEGFA*, etc.	Elite status (proxy for reduced injury)	Elite players had higher mean total gene score (TGS); best *MDR* model: COL5A1 rs12722 + rs3196378 + MIR608 rs4919510 (CC-CC-CC) predictive of elite status.
Rodas et al. 2022 [54]	Spain	FC Barcelona elite footballers	Prospective cohort	N = 46 (24 F, 22 M)	*COL5A1* rs13946, rs16399, rs1134170, rs71746744, rs3196378, rs12722	ACL rupture (history)	Females: rs13946 CC ↑ ACL risk; sex interaction significant; haplotype with rs13946-C overrepresented in injured females.
Collins et al. 2010 [55]	South Africa & Sweden	Recreational and competitive athletes	Case–control (pooled analysis)	350 CL, 126 SD, 41 ATR, 581 controls	*COL1A1* rs1800012 (Sp1-binding site)	Acute soft tissue ruptures (ACL, shoulder dislocation, Achilles tendon)	TT genotype rare but protective. Significantly underrepresented in injury groups vs. controls (e.g., OR = 15 for CL rupture, *p* = 0.0002). Supports TT as protective against acute ruptures.

Legend: ↑ = increased risk, ↓ = decreased risk, OR = Odds Ratio.

**Table 3:** Risk of bias for cohort studies as assessed via NOS criteria.

Study	Sel1	Sel2	Sel3	Sel4	Comp	Out1	Out2	Out3	Total	Risk of Bias
Varamenti et al. 2024 [52]	*		*	*		*	*		5	High
Rodas et al. 2021 [38]	*		*	*	*	*	*	*	7	Low
Del Coso et al. 2022 [37]	*		*	*		*	*	*	6	Moderate
Zhao et al. 2016 [25]	*		*	*	*	*	*	*	7	Low
de Almeida et al. 2022 [40]	*		*	*		*	*	*	6	Moderate
Rodas et al. 2022 [54]	*		*	*	*	*	*	*	7	Low
Clos et al. 2019 [36]	*		*	*	*		*	*	7	Low
Jacob et al. 2022 [51]	*	*	*	*	*	*	*	*	8	Low
Miyamoto-Mikami et al. 2021 [26]	*	*	*	*	*	*	*	*	8	Low

Legend: the NOS domains are abbreviated as Sel1—representativeness of exposed cohort. Sel2—selection of non-exposed cohort. Sel3—ascertainment of exposure. Sel4—demonstration outcome not present at start. Comp—control for major confounders. Out1—assessment of outcome. Out2—was the follow-up long enough? Out3—adequacy of follow-up. * = star given for meeting each criterion.

**Table 4:** Risk of bias in case–control studies as assessed via NOS criteria.

Study	Sel1	Sel2	Sel3	Sel4	Comp	Exp1	Exp2	Exp3	Total	Risk of Bias
Posthumus et al. 2009 [16]	*	*	*	*		*	*	*	7	Low
Raleigh et al. 2009 [22]	*	*	*	*	*	*	*		7	Low
September et al. 2009 [21]	*	*	*	*	*	*	*		7	Low
Collins et al. 2010 [55]	*	*	*	*	*	*	*		7	Low
Malila et al. 2011 [49]	*	*	*	*		*	*		6	Moderate
Perini et al. 2022 [45]	*	*	*	*	*	*	*		7	Low
Massidda et al. 2019 [39]	*	*	*	*	*	*	*		7	Low
Brazier et al. 2025 [43]	*	*	*	*	*	*	*		7	Low
Brazier et al. 2023 [53]		*	*	*		*	*	*	6	Moderate
Gibbon et al. 2017 [48]	*	*	*	*	*	*	*	*	8	Low
Lulińska-Kuklik et al. 2018 [17]	*	*	*	*	*	*	*	*	8	Low
Simunic-Briski et al. 2024 [47]	*	*	*	*	*	*	*	*	8	Low
Brown et al. 2017 [42]	*	*	*	*	*	*	*		7	Low
Posthumus et al. 2009 [41]	*	*	*	*	*	*	*		7	Low
O’Connell et al. 2015 [44]	*	*	*	*	*	*	*		7	Low
Briški et al. 2021 [46]	*	*	*	*	*	*	*		7	Low
McCabe et al. 2018 [50]						*	*		2	High

Legend: the NOS domains are abbreviated as Sel1—case definition adequate, Sel2—representativeness of cases, Sel3—ascertainment of exposure, Sel4—selection of controls, Comp—comparability of cases/controls, Exp1—assessment of exposure, Exp2—same ascertainment method, Exp3—non-response rate, * = star given for meeting each criterion.

## Data Availability

All data collected and used in the review is included in the relevant tables and figures. No new data was created.

## Supplementary Materials

The following supporting information can be downloaded at: https://www.mdpi.com/article/10.3390/genes17020212/s1, PRISMA Checklist.

## References

[1] Hoenig T., Hollander K., Popp K.L., Fredericson M., Kraus E.A., Warden S.J., Tenforde A.S. (2025). International Delphi consensus on bone stress injuries in athletes. Br. J. Sports Med..

[2] Mansfield K., Dopke K., Koroneos Z., Bonaddio V., Adeyemo A., Aynardi M. (2022). Achilles tendon ruptures and repair in athletes—A review of sports-related Achilles injuries and return to play. Curr. Rev. Musculoskelet. Med..

[3] Filbay S.R., Skou S.T., Bullock G.S., Le C.Y., Räisänen A.M., Toomey C., Ezzat A.M., Hayden A., Culvenor A.G., Whittaker J.L. (2022). Long-term quality of life, work limitation, physical activity, economic cost and disease burden following ACL and meniscal injury: A systematic review and meta-analysis for the OPTIKNEE consensus. Br. J. Sports Med..

[4] Saragiotto B.T., Di Pierro C., Lopes A.D. (2014). Risk factors and injury prevention in elite athletes: A descriptive study of the opinions of physical therapists, doctors and trainers. Braz. J. Phys. Ther..

[5] Aicale R., Tarantino D., Maffulli N. (2018). Overuse injuries in sport: A comprehensive overview. J. Orthop. Surg. Res..

[6] Taimela S., Kujala U.M., Osterman K. (1990). Intrinsic risk factors and athletic injuries. Sports Med..

[7] Bulgay C., Çakır V.O., Ergün M.A., Dalkılıç M. (2023). The importance of genetic factors in sports injuries. Innovative Research in Sport Sciences.

[8] Semenova E.A., Hall E.C.R., Ahmetov I.I. (2023). Genes and athletic performance: The 2023 update. Genes.

[9] Maffulli N., Margiotti K., Longo U.G., Loppini M., Fazio V.M., Denaro V. (2013). The genetics of sports injuries and athletic performance. Muscles Ligaments Tendons J..

[10] Varillas-Delgado D., Del Coso J., Gutiérrez-Hellín J., Aguilar-Navarro M., Muñoz A., Maestro A., Morencos E. (2022). Genetics and sports performance: The present and future in the identification of talent for sports based on DNA testing. Eur. J. Appl. Physiol..

[11] Goodlin G.T., Roos A.K., Roos T.R., Hawkins C., Beache S., Baur S., Kim S.K. (2015). Applying personal genetic data to injury risk assessment in athletes. PLoS ONE.

[12] Boden B.P., Sheehan F.T. (2022). Mechanism of non-contact ACL injury: OREF Clinical Research Award 2021. J. Orthop. Res..

[13] Boden B.P., Sheehan F.T., Torg J.S., Hewett T.E. (2010). Noncontact anterior cruciate ligament injuries: Mechanisms and risk factors. J. Am. Acad. Orthop. Surg..

[14] Gianakos A.L., Arias C., Batailler C., Servien E., Mulcahey M.K. (2024). Sex-specific considerations in anterior cruciate ligament injuries in the female athlete: State of the art. J. ISAKOS.

[15] Bahr R., Krosshaug T. (2005). Understanding injury mechanisms: A key component of preventing injuries in sport. Br. J. Sports Med..

[16] Posthumus M., September A.V., Keegan M., O’Cuinneagain D., Van der Merwe W., Schwellnus M.P., Collins M. (2009). Genetic risk factors for anterior cruciate ligament ruptures: *COL1A1* gene variant. Br. J. Sports Med..

[17] Lulińska-Kuklik E., Rahim M., Domańska-Senderowska D., Ficek K., Michałowska-Sawczyn M., Moska W., Kaczmarczyk M., Brzeziański M., Brzeziańska-Lasota E., Cięszczyk P. (2018). Interactions between *COL5A1* gene and risk of the anterior cruciate ligament rupture. J. Hum. Kinet..

[18] Maffulli N., Sharma P., Luscombe K.L. (2004). Achilles tendinopathy: Aetiology and management. J. R. Soc. Med..

[19] Kozlovskaia M., Vlahovich N., Ashton K.J., Hughes D.C. (2017). Biomedical risk factors of Achilles tendinopathy in physically active people: A systematic review. Sports Med.-Open.

[20] Medina Pabón M.A., Naqvi U. (2023). Achilles Tendinopathy. StatPearls.

[21] September A.V., Cook J., Handley C.J., van der Merwe L., Schwellnus M.P., Collins M. (2009). Variants within the *COL5A1* gene are associated with Achilles tendinopathy in two populations. Br. J. Sports Med..

[22] Raleigh S.M., van der Merwe L., Ribbans W.J., Smith R.K., Schwellnus M.P., Collins M. (2009). Variants within the *MMP3* gene are associated with Achilles tendinopathy: Possible interaction with the *COL5A1* gene. Br. J. Sports Med..

[23] Warden S.J., Edwards W.B., Willy R.W. (2021). Preventing bone stress injuries in runners with optimal workload. Curr. Osteoporos. Rep..

[24] da Rocha Lemos Costa T.M., Borba V.Z.C., Correa R.G.P., Moreira C.A. (2022). Stress fractures. Arch. Endocrinol. Metab..

[25] Zhao L., Chang Q., Huang T., Huang C. (2016). Prospective cohort study of the risk factors for stress fractures in Chinese male infantry recruits. J. Int. Med. Res..

[26] Miyamoto-Mikami E., Kumagai H., Tanisawa K., Taga Y., Hirata K., Kikuchi N., Kamiya N., Kawakami R., Midorikawa T., Kawamura T. (2021). Female athletes genetically susceptible to fatigue fracture are resistant to muscle injury: Potential role of COL1A1 variant. Med. Sci. Sports Exerc..

[27] Juginović A., Kekić A., Aranza I., Biloš V., Armanda M. (2025). Next-generation approaches in sports medicine: The role of genetics, omics, and digital health in optimizing athlete performance and longevity—A narrative review. Life.

[28] Ribbans W.J., September A.V., Collins M. (2022). Tendon and ligament genetics: How do they contribute to disease and injury? A narrative review. Life.

[29] Guth L.M., Roth S.M. (2013). Genetic influence on athletic performance. Curr. Opin. Pediatr..

[30] Lv Z.T., Gao S.T., Cheng P., Liang S., Yu S.Y., Yang Q., Chen A.M. (2017). Association between polymorphism rs12722 in *COL5A1* and musculoskeletal soft tissue injuries: A systematic review and meta-analysis. Oncotarget.

[31] Yang N., MacArthur D.G., Gulbin J.P., Hahn A.G., Beggs A.H., Easteal S., North K. (2003). ACTN3 genotype is associated with human elite athletic performance. Am. J. Hum. Genet..

[32] Liu J., Cai W., Zhang H., He C., Deng L. (2013). Rs143383 in the growth differentiation factor 5 (*GDF5*) gene is significantly associated with osteoarthritis (OA): A comprehensive meta-analysis. Int. J. Med. Sci..

[33] Guo R., Aizezi A., Fan Y., Ji Z., Li W., Li Y., Wang Z., Ning K. (2022). Association between matrix metalloproteinase-3 gene polymorphisms and tendon-ligament injuries: Evidence from a meta-analysis. BMC Sports Sci. Med. Rehabil..

[34] Page M.J., McKenzie J.E., Bossuyt P.M., Boutron I., Hoffmann T.C., Mulrow C.D., Shamseer L., Tetzlaff J.M., Akl E.A., Brennan S.E. (2021). The PRISMA 2020 statement: An updated guideline for reporting systematic reviews. BMJ.

[35] (2023). Covidence Systematic Review Software.

[36] Clos E., Pruna R., Lundblad M., Artells R., Esquirol Caussa J. (2019). ACTN3 single nucleotide polymorphism is associated with non-contact musculoskeletal soft-tissue injury incidence in elite professional football players. Knee Surg. Sports Traumatol. Arthrosc..

[37] Del Coso J., Rodas G., Buil M.Á., Sánchez-Sánchez J., López P., González-Ródenas J., Gasulla-Anglés P., López-Samanes Á., Hernández-Sánchez S., Iztueta A. (2022). Association of the ACTN3 rs1815739 polymorphism with physical performance and injury incidence in professional women football players. Genes.

[38] Rodas G., Moreno-Pérez V., Del Coso J., Florit D., Osaba L., Lucia A. (2021). Alpha-actinin-3 deficiency might affect recovery from non-contact muscle injuries: Preliminary findings in a top-level soccer team. Genes.

[39] Massidda M., Voisin S., Culigioni C., Piras F., Cugia P., Yan X., Eynon N., Calò C.M. (2019). ACTN3 R577X polymorphism is associated with the incidence and severity of injuries in professional football players. Clin. J. Sport Med..

[40] de Almeida K.Y., Cetolin T., Marrero A.R., Aguiar Junior A.S., Mohr P., Kikuchi N. (2022). A pilot study on the prediction of non-contact muscle injuries based on ACTN3 R577X and ACE I/D polymorphisms in professional soccer athletes. Genes.

[41] Posthumus M., September A.V., O’Cuinneagain D., van der Merwe W., Schwellnus M.P., Collins M. (2009). The *COL5A1* gene is associated with increased risk of anterior cruciate ligament ruptures in female participants. Am. J. Sports Med..

[42] Brown K.L., Seale K.B., El Khoury L.Y., Posthumus M., Ribbans W.J., Raleigh S.M., Collins M., September A.V. (2017). Polymorphisms within the *COL5A1* gene and regulators of the extracellular matrix modify the risk of Achilles tendon pathology in a British case-control study. J. Sports Sci..

[43] Brazier J., Antrobus M.R., Callus P.C., Herbert A.J., Stebbings G.K., Martin D., Day S.H., Kilduff L.P., Bennett M.A., Erskine R.M. (2025). Variants within the *MMP3* and *COL5A1* genes associate with soft tissue injury history in elite male rugby athletes. J. Sci. Med. Sport.

[44] O’Connell K., Knight H., Ficek K., Leonska-Duniec A., Maciejewska-Karlowska A., Sawczuk M., Stepien-Slodkowska M., O’Cuinneagain D., van der Merwe W., Posthumus M. (2015). Interactions between collagen gene variants and risk of anterior cruciate ligament rupture. Eur. J. Sport Sci..

[45] Perini J.A., Lopes L.R., Guimarães J.A.M., Goes R.A., Pereira L.F.A., Pereira C.G., Mandarino M., Villardi A.M., de Sousa E.B., Cossich V.R.A. (2022). Influence of type I collagen polymorphisms and risk of anterior cruciate ligament rupture in athletes: A case-control study. BMC Musculoskelet. Disord..

[46] Briški N., Vrgoč G., Knjaz D., Janković S., Ivković A., Pećina M., Lauc G. (2021). Association of the matrix metalloproteinase 3 (MMP3) single nucleotide polymorphisms with tendinopathies: A case–control study in high-level athletes. Int. Orthop..

[47] Simunic-Briski N., Vrgoč G., Knjaz D., Janković S., Dembic Z., Lauc G. (2024). MMP3 single-nucleotide polymorphisms are associated with noncontact ACL injuries in competing high-level athletes. J. Orthop. Res..

[48] Gibbon A., Hobbs H., van der Merwe W., Raleigh S.M., Cook J., Handley C.J., Posthumus M., Collins M., September A.V. (2017). The *MMP3* gene in musculoskeletal soft tissue injury risk profiling: A study in two independent sample groups. J. Sports Sci..

[49] Malila S., Yuktanandana P., Saowaprut S., Jiamjarasrangsi W., Honsawek S. (2011). Association between matrix metalloproteinase-3 polymorphism and anterior cruciate ligament ruptures. Genet. Mol. Res..

[50] McCabe K., Collins C. (2018). Can genetics predict sports injury? The association of the genes *GDF5*, *AMPD1*, *COL5A1* and *IGF2* on soccer player injury occurrence. Sports.

[51] Jacob Y., Anderton R.S., Cochrane Wilkie J.L., Rogalski B., Laws S.M., Jones A., Spiteri T., Hince D., Hart N.H. (2022). Genetic variants within *NOGGIN, COL1A1, COL5A1*, and *IGF2* are associated with musculoskeletal injuries in elite male Australian Football League players: A preliminary study. Sports Med.-Open.

[52] Varamenti E., Pullinger S.A., Kollias P., Chini V. (2024). Identification of specific injury-related SNPs in high-level athletes of Arab origin: A pilot study. Heliyon.

[53] Brazier J., Antrobus M.R., Herbert A.J., Callus P.C., Khanal P., Stebbings G.K., Day S.H., Heffernan S.M., Kilduff L.P., Bennett M.A. (2023). Gene variants previously associated with reduced soft-tissue injury risk: Part 2—Polygenic associations with elite status in rugby. Eur. J. Sport Sci..

[54] Rodas G., Cáceres A., Ferrer E., Balagué-Dobón L., Osaba L., Lucia A., González J.R. (2022). Sex differences in the association between risk of anterior cruciate ligament rupture and *COL5A1* polymorphisms in elite footballers. Genes.

[55] Collins M., Posthumus M., Schwellnus M.P. (2010). The *COL1A1* gene and acute soft tissue ruptures. Br. J. Sports Med..

[56] Wells G.A., Shea B., O’Connell D., Peterson J., Welch V., Losos M., Tugwell P. (2011). The Newcastle–Ottawa Scale (NOS) for Assessing the Quality of Nonrandomised Studies in Meta-Analyses.

[57] Miyamoto-Mikami E., Miyamoto N., Kumagai H., Hirata K., Kikuchi N., Zempo H., Kimura N., Kamiya N., Kanehisa H., Naito H. (2019). *COL5A1* rs12722 polymorphism is not associated with passive muscle stiffness and sports-related muscle injury in Japanese athletes. BMC Med. Genet..

[58] Gkritzali E., Akam L., Mastana S. (2024). Role of *COL5A1* gene polymorphism (rs12722) in lower limb musculoskeletal injuries among adults: A systematic review and meta-analysis. J. Res. Pract. Musculoskelet. Syst..

[59] Gutiérrez-Hellín J., Baltazar-Martins G., Aguilar-Navarro M., Ruiz-Moreno C., Oliván J., Del Coso J. (2021). Effect of ACTN3 R577X genotype on injury epidemiology in elite endurance runners. Genes.

[60] Guo R., Ji Z., Gao S., Aizezi A., Fan Y., Wang Z., Ning K. (2022). Association of *COL5A1* gene polymorphisms and musculoskeletal soft tissue injuries: A meta-analysis based on 21 observational studies. J. Orthop. Surg. Res..

[61] Kania K., Colella F., Riemen A.H.K., Wang H., Howard K.A., Aigner T., Dell’ACcio F., Capellini T.D., Roelofs A.J., De Bari C. (2020). Regulation of *Gdf5* expression in joint remodelling, repair and osteoarthritis. Sci. Rep..

[62] Contrò V., Schiera G., Abbruzzo A., Bianco A., Amato A., Sacco A., Macchiarella A., Palma A., Proia P. (2018). An innovative way to highlight the power of each polymorphism on elite athletes phenotype expression. Eur. J. Transl. Myol..

